# Development and validation of a 3D printed phantom for image quality assessment in fluoroscopy

**DOI:** 10.1002/acm2.70650

**Published:** 2026-06-04

**Authors:** Túlio Guilherme Soares Marques, Diana Rodrigues de Pina

**Affiliations:** ^1^ Institute of Bioscience São Paulo State University (UNESP) Botucatu São Paulo Brazil; ^2^ Department of Tropical Diseases and Imaging Diagnosis, Faculty of Medicine of Botucatu São Paulo State University (UNESP) Botucatu São Paulo Brazil

**Keywords:** 3D‐printed phantom, fluoroscopy, image quality, interventional radiology, quality control

## Abstract

**Background:**

Low‐contrast detectability is a key image‐quality parameter in fluoroscopy, directly influencing diagnostic accuracy and patient safety. Commercial phantoms such as the CDRAD provide reliable assessments but are costly and lack flexibility for routine use.

**Purpose:**

To develop and validate a low‐cost 3D‐printed phantom for fluoroscopy quality control, designed with low‐contrast performance similar to the commercial reference standard CDRAD, and to offer a reproducible framework for clinical implementation. This represents an original and accessible strategy for systematic fluoroscopy image‐quality assessment.

**Methods:**

The proposed 3D‐printed low‐contrast phantom (hereafter referred to as the LC phantom) was modeled in a Computer‐Aided Design (CAD) software and printed in polylactic acid (PLA) with 100% infill. It features a modular circular design adaptable to different fields of view. Quantitative validation included correlation between theoretical subject contrast (SC) and image‐based contrast‐to‐noise ratio (CNR), with comparisons to the CDRAD under clinically representative beam qualities (65–100 kVp), using additional copper filtration to produce hardened X‐ray spectra typical of fluoroscopic practice. Subjective comparison of both phantoms was performed through multicenter testing on four fluoroscopy systems (three mobile and one fixed).

**Results:**

CNR increased consistently with hole depth for both phantoms across all beam qualities, with linear regression models yielding coefficients of determination (R^2^) ranging from 0.88 to 0.99. Visual evaluations yielded mean low‐contrast thresholds of 3.11 ± 0.26%, 2.66 ± 0.31%, and 2.70 ± 0.27% for 65, 80, and 100 kVp, respectively, with a maximum deviation of 0.10 SC units relative to the reference phantom, indicating reproducible and consistent performance across the evaluated beam qualities.

**Conclusions:**

The proposed LC phantom is a practical and affordable tool for routine fluoroscopy image quality assessment. Its methodology can be adapted to different beam qualities and institutional protocols, supporting harmonized and accessible image‐quality assessment in clinical practice.

## 1 INTRODUCTION

Fluoroscopy enables real‐time visualization of internal structures and is widely used in interventional procedures.^1^ However, the quality of the produced image directly influences diagnostic accuracy and patient safety, making it essential to implement rigorous quality control (QC) protocols to ensure proper calibration and operation of radiological equipment.^2^, ^3^ Organizations such as the American Association of Physicists in Medicine (AAPM), the International Electrotechnical Commission (IEC), and the International Atomic Energy Agency (IAEA) establish guidelines for the systematic evaluation of critical image quality parameters, including low‐contrast resolution.^4^, ^5^, ^6^

Low‐contrast resolution refers to the ability of an imaging system to distinguish objects or structures that exhibit only minimal differences in X‐ray attenuation relative to their surrounding background.^7^ Depending on the application, phantoms may be designed to evaluate either positive or negative contrast structures. Positive‐contrast configurations employ high‐attenuation inserts embedded in a uniform background, while negative‐contrast designs rely on air cavities within a solid matrix, generating features of lower attenuation relative to their surroundings. The latter approach is particularly suitable for fluoroscopic imaging, where low‐density structures often simulate the soft tissue contrast conditions encountered in clinical practice.

Commonly used commercial phantoms for low‐contrast resolution assessment in fluoroscopy include the FLUKE® R/F QC Phantom 07–647, the CIRS® 903 Radiography/Fluoroscopy QA Phantom, and the ARTINIS® CDRAD.^8^, ^9^, ^10^ They are widely adopted in clinical practice and are consistent with established international QC standards for fluoroscopy systems, such as IEC 61223‐3‐1, and each offers specific advantages regarding manufacturing complexity, reproducibility, and sensitivity to noise. Table 1 provides a comparison based on the range of low‐contrast structures, physical dimensions, weight and a market price estimation.

**TABLE 1:** Comparison of commercial image‐quality phantoms commonly used for low‐contrast resolution performance in fluoroscopy. The table summarizes the range of low‐contrast structures, physical dimensions, weight and market price estimation (USD).

Phantom	Low contrast structures	Phantom size and weight	Market price estimation (USD)
FLUKE ® R/F QC Phantom 07–647	Four circular low‐contrast objects with variable diameters (2, 4, 6, and 8 mm); object thickness is constant.	17.78 cm x 17.17 cm x 1.42 cm/ 0.5 kg	550–700
CIRS ® 903 Radiography/Fluoroscopy QA Phantom	Cylindrical holes with fixed diameter (9.53 mm) and hole depths ranging from 1.73 mm to 0.10 mm.	25.4 cm x 25.4 cm x 20.3 cm / 16.8 kg (complete set)	1500–5000
ARTINIS ®CDRAD	Contrast‐detail matrix of acrylic and air, ranging from 0.3‐8.0 mm of diameter and 0.3‐8.0 mm of depth	26.4 cm x 26.4 cm x 0.76 cm / 1.34 kg	4000–8000

Despite their proven reliability, these phantoms present notable limitations in routine clinical implementation. The Fluke ® R/F QC Phantom (Model 07–647) provides only a limited low‐contrast challenge, offering a small set of discrete masses that restricts its sensitivity to subtle variations in contrast performance. The CIRS ® Model 903, although comprehensive for general radiography and fluoroscopy QC, relies on a uniform PMMA‐equivalent epoxy background and fixed aluminum contrast‐detail structures, which limits its ability to represent realistic anatomical noise and the heterogeneous attenuation conditions typical of fluoroscopic imaging. The Artinis ® CDRAD, on the other hand, was originally designed for radiographic systems rather than for dynamic fluoroscopy. As a result, its fixed matrix geometry reflects contrast–detail relationships optimized for static, higher‐dose imaging conditions.

There is a need for a more affordable and dynamic device that retains effective diagnostic capability while allowing straightforward comparison with validated phantoms. Unlike conventional static designs, this system should incorporate interchangeable modules or inserts to enable an adaptable approach to low‐contrast assessment. Furthermore, it must be adaptable to diverse imaging systems, thereby ensuring consistent QC practices even in resource‐limited settings. Recent advances in 3D printing have enabled the rapid prototyping of customized phantoms with high geometric precision and reproducibility, providing a cost‐effective alternative to traditional commercial devices.^11^

This study aimed to design, fabricate, and validate a 3D‐printed phantom for fluoroscopy QC, specifically intended to evaluate low‐contrast resolution performance. This development addresses practical implementation challenges while preserving diagnostic rigor, providing a quantitatively validated alternative that supports consistent image quality assessment across diverse clinical environments.

## 2 METHODS AND MATERIALS

The low‐contrast phantom (LC) was developed through planning, printing, and assembling, as detailed in Section 2.1, and validation, as detailed in Section 2.2.

### 2.1 Planning, printing and assembling

The conceptual development and construction of the phantom are presented in modular form, corresponding to the 4.5″, 6.5″, and 9″ fluoroscopic fields of view (FOVs). Modules were designed to provide a low‐contrast challenge at each geometric condition imaged. The overall design employs three simple, interlocking modules to facilitate handling, transport, and routine clinical use, while preserving consistent alignment across different field configurations.

**FIGURE 1:**
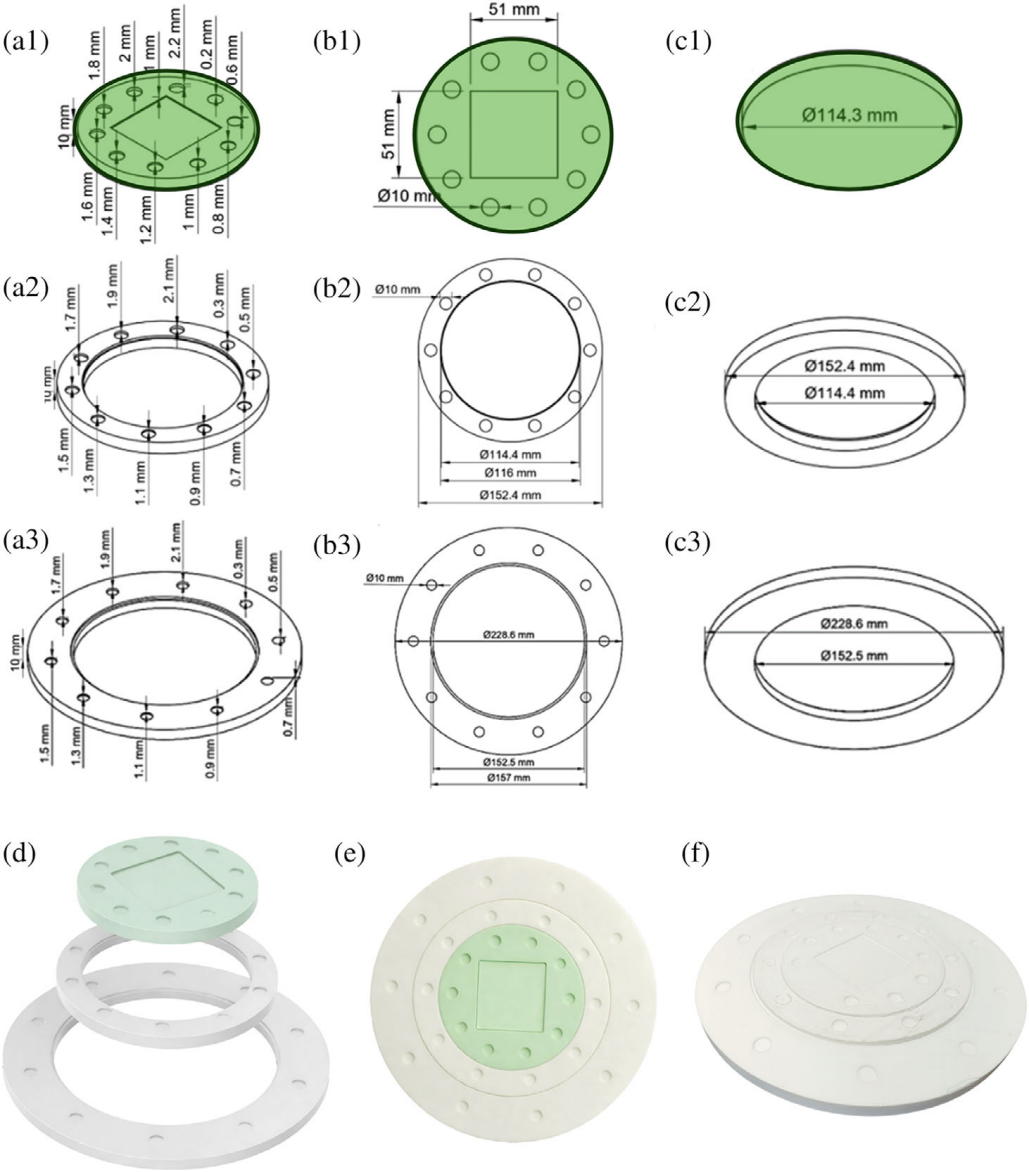
Design and assembly of the modular LC phantom. Panels a–c illustrate the three target modules designed. Panels a1–a3 show the detailed isometric cut views illustrating the spatial layout and depth distribution of the low‐contrast structures. Panels b1–b3 show the corresponding front views of the inserts. Panels c1–c3 depict cross‐sectional views of the assembled phantom. Panel d shows a computer‐generated rendering of the phantom (Fusion 360, version 2023.2.1, Autodesk Inc., San Rafael, CA, USA), while panels e and f display photographic images of the assembly process and the fully assembled phantom. In panel e, the central region was slightly processed for visualization purposes to match the highlighted area. The green‐highlighted part represents the central module, which receives the interlocks of the larger modules.

An integrated schematic and structural overview of the phantom is presented in Figure 1, which includes three complementary sets of views for each module: isometric cut views illustrating the spatial layout and depth distribution of the low‐contrast structures (panels A1–A3); top views with physical dimensions (panels B1–B3), and cross‐sectional views showing the external geometry (panels C1–C3). Panels D–F display the modular architecture and assembly process, including the exploded view (D), a top‐down view of the assembled phantom (E), and an isometric perspective of the final configuration (F). The green‐highlighted parts represent the central module, which serves as the core structure and receives the interlocking interfaces of the larger modules. The interlocking modules are displayed in white.

In the planning phase, the parts were designed using a commercially available Computer‐Aided Design (CAD) software (Fusion 360, version 2023.2.1, Autodesk Inc., San Rafael, CA, USA), which offers free educational licensing. The phantom was designed with a circular geometry to reflect the typical FOV of image intensifier (II) systems used in fluoroscopy. Although flat‐panel detectors are rectangular, this design remains compatible without limiting positioning or alignment. The circular design also facilitates reproducible alignment with the fluoroscopic imaging field, avoiding misalignment between the phantom and the collimated field during repeated acquisitions.^12^ In addition, since fluoroscopic systems operate with different FOV sizes depending on the magnification mode, the phantom was designed to allow modular use in multiple configurations.

The first module (plate A) was designed for a 4.5″ FOV, and includes a low‐contrast structure range spanning 0.2–2.2 mm with 0.2 mm steps, since the X‐ray beam is more intense and uniform near the central region of the II or flat‐panel detector, where geometric distortion and peripheral signal losses are minimized. This configuration, as well as those of the larger modules, was empirically tested during prototyping and consistently provided the best performance across beam qualities for this FOV. As a result, it was adopted to ensure realistic, dose‐ and geometry‐dependent variations in low‐contrast performance typically observed in clinical fluoroscopy systems. The central module was also symmetrically designed to allow alignment verification between the X‐ray field and detector center, although this feature was not included in the current validation.

The second plate (plate B) was designed for a 6″ FOV. The second module preserves the overall structural characteristics of the first module but incorporates an adjusted range of hole depths (0.3–2.1 mm), shifted toward slightly higher values to maintain an equivalent low‐contrast detectability under the reduced beam fluence and increased scatter conditions of larger FOVs. Similar to the central module, this configuration was empirically tested during the prototype phase and showed improved sensitivity for low‐contrast structures at this field size. Additionally, because its depth increments are offset by 0.1 mm relative to the central module, it introduces complementary contrast levels, offering a broader range of detection challenges.

The third plate (plate C) was designed for a 9″ FOV. The third module preserves the adjusted range of hole depths of the second module.

All modules contain ten targets with identical diameters, arranged in a circular pattern to ensure homogeneous irradiation along the radial axis of the X‐ray beam. This design standardizes the irradiation geometry and reduces scatter‐related artifacts, improving measurement reproducibility.

A negative low‐contrast design was selected because structures defined by material holes require less material and simplify manufacturing. Different hole depths and spatial distributions were tested to define a configuration with an appropriate dynamic range for negative low‐contrast detectability under clinically relevant conditions. The selected configuration consistently provided the most informative spread of low‐contrast structures while preserving manufacturability and cost‐effectiveness.

All holes were fabricated with a 10‐mm diameter, a dimension established through experimental testing to minimize edge artifacts and ensure high measurement stability, thereby supporting consistent low‐contrast assessment and precise ROI delineation.

The overall plate thickness was likewise set to 10 mm, providing adequate mechanical rigidity while accommodating the full range of required hole depths. This specification was limited by the 0.1‐mm z‐axis resolution of commercial 3D printers, ensuring reliable depth increments without compromising structural integrity. Since low‐contrast detectability depends primarily on attenuation differences and noise behavior, these dimensions remain fully aligned with those used in established contrast–detail and low‐contrast phantoms for fluoroscopy.^13^

The phantom components were fabricated using a Creality ®Ender 6 fused deposition modeling (FDM) 3D printer. Model slicing was performed with the open‐source software UltiMaker Cura ® (version 5.8). Polylactic acid (PLA) filament was selected as the printing material due to its low cost, wide availability, and sufficient mechanical stability for use in QC applications.^14^ Printing was carried out with a layer height of 0.2 mm, a 0.4 mm nozzle, a print speed of 60 mm/s, a nozzle temperature of 200°C, 100% infill, and a build plate temperature of 60°C. Those conditions guarantee a reproducibility of 0.1 mm in PLA thickness.^15^

The final assembly of the phantom was achieved through a simple interlocking mechanism between modular components, ensuring ease of handling and rapid setup during routine QC procedures. Supplementary File S1 contains the STL files used for phantom fabrication, which is also publicly available through the Zenodo repository (DOI: 10.5281/zenodo.18827666).

### 2.2 Validation

A validation process was conducted to ensure that the LC phantom provides physically consistent, quantitatively traceable, and clinically meaningful measurements when compared with a reference low‐contrast phantom, namely the Artinis® CDRAD. The CDRAD phantom was selected due to its established role in contrast–detail analysis image quality evaluation, where it provides a structured and reproducible matrix of contrast–thickness combinations widely used in QC studies.^10^ Although originally developed for radiographic systems, its standardized contrast–detail geometry offers a suitable and well‐characterized reference framework for comparative validation. Accordingly, the phantom was required to demonstrate consistent low‐contrast performance across clinically relevant beam qualities, with reproducible visual assessments across observers and systems.

Within this framework, the primary output parameter was defined as the subject contrast (SC) threshold. The assessment strategy combined quantitative and visual approaches. Quantitatively, contrast‐to‐noise ratio (CNR) measurements were obtained using the MicroDicom software (version 2025.3)^16^ to establish a relationship between image‐based detectability and theoretical SC values, enabling the determination of SC‐equivalent thresholds. Visually, experienced observers independently identified the limiting detectable target under standardized viewing conditions, allowing determination of the corresponding SC. Dosimetric calibration information was incorporated through the use of a calibrated multimeter (RTI ® Piranha).

**FIGURE 2:**
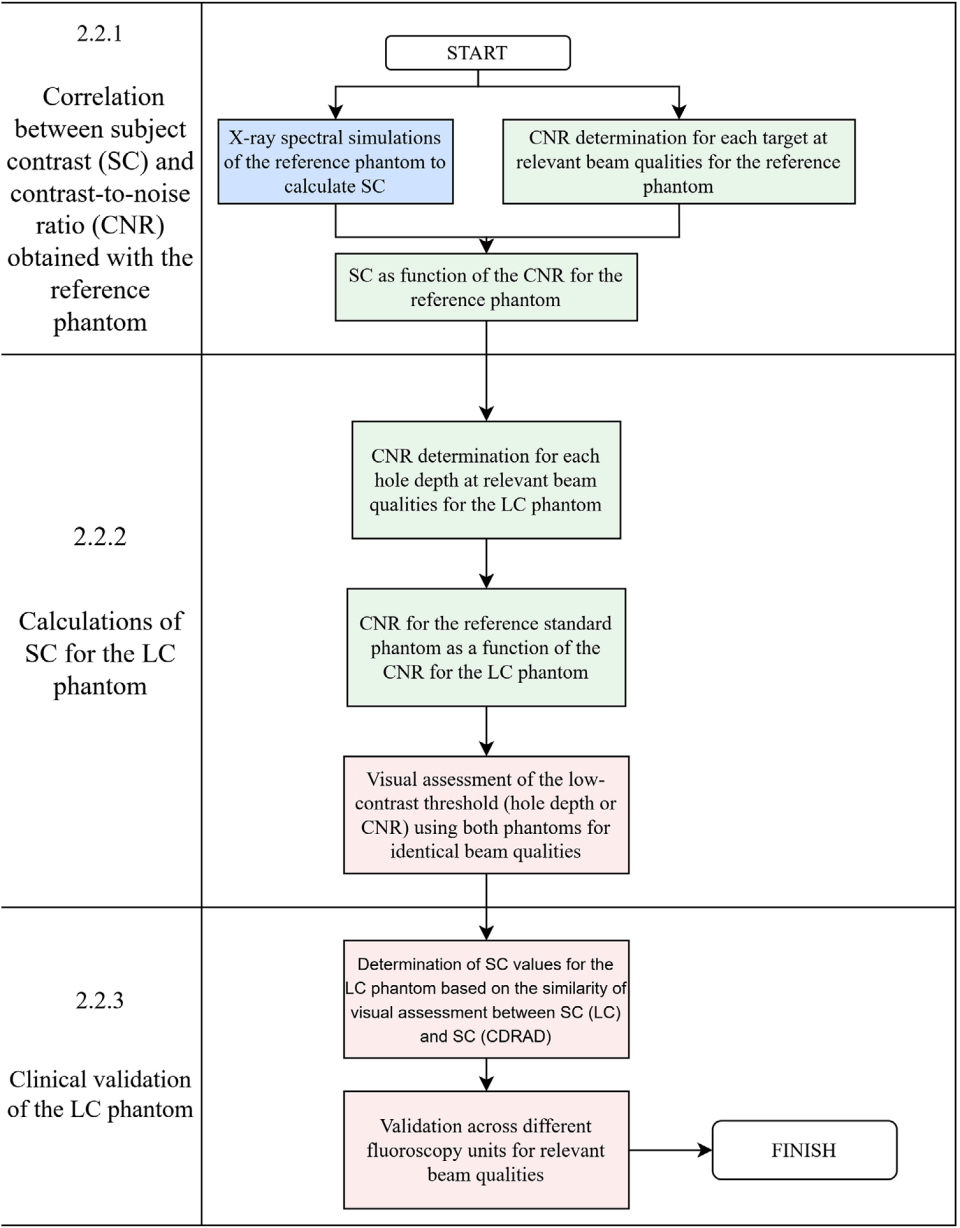
Validation workflow for the proposed LC phantom. Blue boxes represent computational simulation steps. Green boxes represent quantitatively measured experimental data obtained from fluoroscopic acquisitions and CNR analysis. Pink boxes represent data collected through qualitative visual assessment.

The validation workflow illustrated in Figure 2 summarizes the stepwise strategy adopted to establish the equivalence between the proposed LC phantom and the reference standard CDRAD phantom. Blue boxes represent simulation‐based steps, green boxes correspond to quantitatively measured experimental data, and pink boxes indicate visually assessed data.

In Stage 2.2.1, X‐ray spectral simulations were performed using the SpekPy library to calculate theoretical SC values for each hole depth and beam quality for the CDRAD. These values were then linked to experimentally measured CNR values obtained from fluoroscopic images, establishing a reference SC–CNR relationship for the CDRAD. A detailed description of the spectral simulation platform and modeling methodology is provided in the subsequent sections.

In Stage 2.2.2, fluoroscopic images of the proposed LC phantom were acquired using the technique hold feature, which maintained the same beam qualities across acquisitions. A standardized acquisition geometry was used, and CNR values were calculated for each target depth. The SC–CNR relationship established for the CDRAD in Stage 2.2.1 was then used to convert the measured CNR values of the LC phantom into CDRAD‐traceable SC‐equivalent values. This conversion was performed across the relevant depth range, without direct one‐to‐one matching between geometrically identical holes. Fluoroscopic images were exported directly from the imaging system in uncompressed DICOM format to preserve original pixel values for CNR analysis.

In Stage 2.2.3, a qualitative validation was performed through a visual assessment of low‐contrast detectability. Three experienced medical physicists and two radiologists independently evaluated images from both phantoms. Image acquisition was performed using clinically relevant beam qualities, consistent exposure technique and dose rate settings, minimum magnification mode, and standardized geometric positioning for both phantoms. Visual assessment was conducted on a calibrated high‐resolution medical‐grade monitor under controlled ambient lighting conditions. Observers were asked: *“What is the last hole you can still reliably visualize?*”, identifying the smallest visible contrast threshold target for each phantom across multiple fluoroscopy systems and clinically relevant beam qualities. Following this equivalence validation, the LC phantom underwent multi‐system evaluation across three additional fluoroscopy units (two mobile and one fixed), using the same beam quality and standardized acquisition conditions. This phase assessed reproducibility and clinical applicability across heterogeneous systems, aiming precisely at possible variability in the phantom's response in relation to the equipment profile (X‐ray spectrum and detector).

For each system, the same three medical physicists and two radiologists independently performed visual threshold identification under identical viewing conditions, again answering the question: *“What is the last hole you can still reliably visualize?”*. Interobserver variability was quantified as the standard deviation of the corresponding SC threshold values.

#### 2.2.1 Correlation between SC and CNR obtained with the CDRAD phantom

International QC protocols for fluoroscopy systems report low‐contrast acceptability thresholds in the order of a few percent (typically between 2 and 4%), in terms of SC thresholds, depending on detector technology and beam conditions.^17^

It is crucial to distinguish between SC and image contrast. The first term refers to the relative difference in transmitted X‐ray intensity between a structure and its surrounding background prior to detection and is determined by material properties, thickness, and beam quality. Image contrast represents the contrast observed in the final displayed image and is influenced by detector response, quantum noise, system transfer characteristics, and post‐processing algorithms.^18^ In fluoroscopy, where automatic exposure control and real‐time image processing are routinely applied, the displayed image contrast does not directly correspond to the underlying SC.

For the CDRAD, SC can be theoretically computed using X‐ray spectral simulations, given its well‐defined geometry and material properties; however, this quantity cannot be directly extracted from fluoroscopic images. The pixel values in fluoroscopy represent processed relative intensities rather than absolute fluence, and therefore depend on multiple system‐specific factors, including detector response, automatic noise reduction, filtration, pulse rate, and post‐processing algorithms. Consequently, the object–background contrast observed in the final image no longer reflects the theoretical SC, but instead a composite result influenced by system noise, image processing, and exposure conditions.^12^, ^18^

To compare theoretical SC values with fluoroscopic image contrast, a quantitative metric extractable from image data is required. CNR provides the essential link between theoretical contrast values and image‐based measurements, allowing the theoretically computed SC values to be translated into an image‐based measure of detectability. By normalizing the difference between object and background signal to background noise, CNR offers a robust descriptor of low‐contrast performance that reflects the combined influence of contrast, noise, and system‐dependent imaging characteristics.

To compare image contrast on the proposed phantom and the CDRAD, three clinically representative beam qualities, described in Table 2, were selected based on specific combinations of peak tube voltage (kVp), additional copper filtration (mm Cu), focus–detector distance (FDD), and half‐value layer (HVL). Those beams correspond to commonly used fluoroscopic operating modes in which copper filtration is progressively increased with tube voltage to reduce patient entrance dose while maintaining adequate beam hardening under typical clinical conditions.^19^, ^20^ Consequently, the selected beam qualities reflect realistic clinical exposure scenarios and provide a robust and appropriate basis for evaluating the low‐contrast performance of the proposed device.^21^, ^22^

**TABLE 2:** Technical specifications of the selected beam qualities (Q1–Q3), including tube potential (kVp), additional copper filtration, focus‐to‐detector distance (FDD) and measured half‐value layer (HVL).

Parameter	Q1	Q2	Q3
kVp	65	80	100
Additional copper filtration (mm)	0.3	0.6	0.9
FDD (mm)	1000		
HVL (mm Al)	5.6	8.1	10.0

To establish a quantitative relationship between SC and image‐derived CNR, the first step was to calculate the SC for the CDRAD. Theoretical SC values for each hole depth and beam quality were computed using the SpekPy library,^23^ in Python, which enabled the X‐ray spectral modeling given tube voltage, filtration, phantom material, and geometry parameters described in Table 2. For a fixed beam quality, SC was calculated according to Equation (1):

(1) $$SC\;{\left(\%\right)}_{CDRAD}=\frac{\Psi_{Hole\;depth}-\Psi_{Backgroud}}{\Psi_{Background}}\;x\;100,$$

where $\Psi_{Hole\;depth}$ corresponds to the transmitted photon fluence through the low‐contrast structure, calculated as the integral of the spectral photon fluence distribution as a function of energy, and $\Psi_{Background}$ corresponds to the transmitted photon fluence through the 10 mm PMMA background, obtained from the same integration procedure.^18^

**FIGURE 3:**
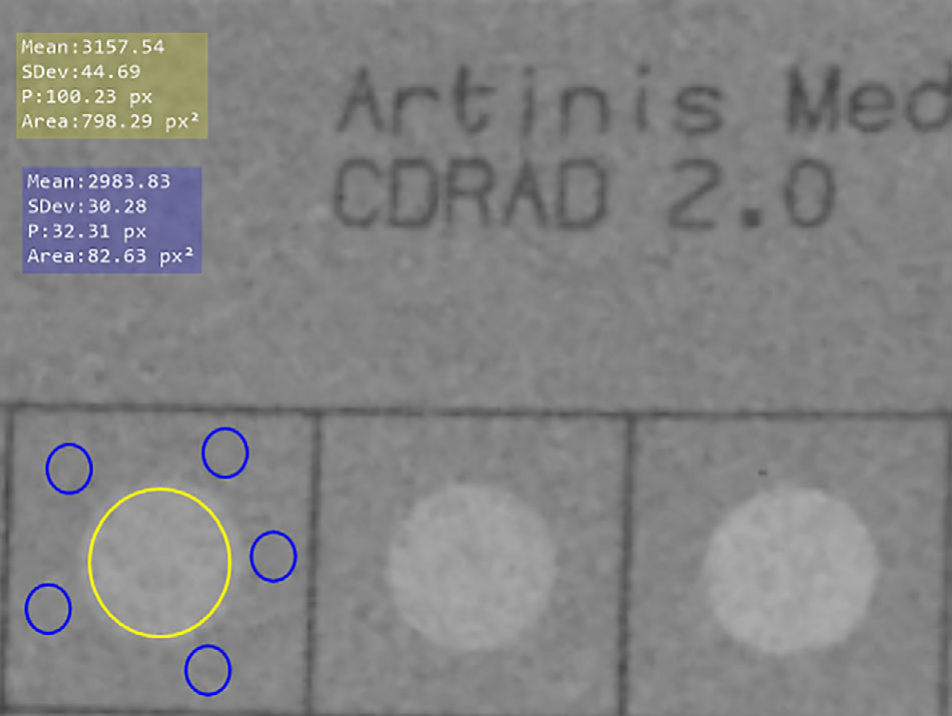
Positioning of ROIs used for CNR determination in the CDRAD. The yellow ROI indicates the region used to calculate $MPV_{Hole\;depth}$, while the blue ROIs correspond to the regions used to calculate $MPV_{Background}$. In this example, CNR was calculated for the 4.0‐mm hole depth. For clarity, only one representative ROI position is shown for the $MPV_{Hole\;depth}$ measurement.

From images acquired under the same beam quality used in the theoretical SC calculations, CNR was determined for each CDRAD 8‐mm‐diameter low‐contrast hole using Equation (2):

(2) $$CNR_{Hole\;depth}=\frac{\left|MPV_{Hole\;depth}-MPV_{Background}\right|}{STD_{Background}},$$

where $MPV_{\mathrm{Hole}\;\mathrm{depth}}$ is the mean pixel value (MPV) within the low‐contrast hole, $MPV_{\mathrm{Background}}$ is the MPV in a background region outside the target hole, and $STD_{\mathrm{Background}}$ is the standard deviation of pixel values in that same uniform background area.^5^ Two values of $MPV_{\mathrm{Hole}\;\mathrm{depth}}$ and five values of $STD_{\mathrm{Background}}$ were computed for each hole depth, and the mean of these values was used for the final calculation. The CNR was calculated using the open‐source software MicroDicom DICOM Viewer (version 2025.3),^16^ employing circular regions of interest (ROI) which fully covered the 8‐mm‐diameter low‐contrast target, displayed in a EIZO © RadiForce RX340 monitor within the radiology department, which met all QC criteria. The 8‐mm diameter structures were selected as they provide sufficiently large and uniform ROIs to ensure stable mean pixel value estimation and robust noise characterization under fluoroscopic conditions. Figure 3 illustrates ROI positioning on the CDRAD.

Therefore, a linear relationship between SC (simulated) and CNR (measured) was established for the CDRAD for beam qualities Q1‐Q3, as described in Equation (3):

(3) $$SC_{Hole-CDRAD}\;\left(\%\right)=a*CNR_{Hole-CDRAD}+b,$$

where a and b represent the slope and intercept of the linear interpolation, respectively.

The association process between SC and CNR used a mobile General Electric ® OEC One system, hereby denoted Unit A, for the three beam qualities. All images were acquired following the exact same imaging geometry, in continuous mode, with default noise reduction configuration (denoise 2) and an anti‐scatter grid in place. Prior to data acquisition, Unit A was verified to be fully compliant with all routine QC tests. Using a single fluoroscopy unit for all quantitative measurements ensured that the images of both the CDRAD and the LC phantom were obtained under identical conditions. HVL measurements were directly performed using a properly calibrated RTI ® Piranha multimeter. The instrument was calibrated in terms of air kerma at an accredited secondary standard dosimetry laboratory. The standardized RQR beam qualities used for calibration encompassed the diagnostic energy range investigated in this study.

#### 2.2.2 Calculations of the SC for the LC phantom

The second stage of validation aimed to establish a relationship between the theoretical SC values simulated for the CDRAD and the CNR obtained with the LC phantom. While the CDRAD is composed of polymethyl methacrylate (PMMA) and the LC phantom was manufactured using PLA, both simulators present similar target hole depths (0.3–2.5 mm).

Although SC can be computed for the CDRAD's targets, it cannot be directly derived for the LC, since SpekPy does not currently support by default the definition of 3D‐printed materials such as PLA, whose density differs from standard PMMA. Simulating a material with incorrect attenuation properties would directly affect the calculated SC (Equation 1), as it depends on the transmitted photon fluence, which in turn is governed by the energy‐dependent attenuation behavior of the material for a given X‐ray spectrum.^18^ Because CNR varies linearly with hole depth in both phantoms, which span comparable depth ranges, a direct linear relationship can be established between $CNR_{Hole\;depth-LC}$ and $CNR_{Hole\;depth-CDRAD}$ for each beam quality (Equation (4)):

(4) $$CNR_{Hole\;depth-LC}=c*CNR_{Hole\;depth-CDRAD}+d,$$

where coefficients c and d correspond to the slope and intercept of the linear regression for each beam quality. Each point represents paired CNR values measured for the same hole depth in both phantoms (e.g., 0.8, 1.0, 1.6, and 2.0 mm). This formulation provides the quantitative basis for translating CNR measurements obtained with the LC phantom into a CDRAD‐traceable reference space.

**FIGURE 4:**
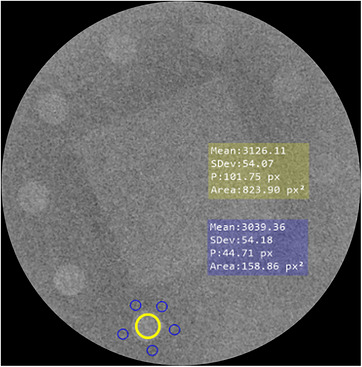
Positioning of ROIs used for CNR determination in the LC phantom. The yellow ROI indicates the region used to calculate $MPV_{Hole\;depth}$, while the blue ROIs correspond to the regions used to calculate $MPV_{Background}$. In this example, CNR was calculated for the 2.2‐mm hole depth. For clarity, only one representative ROI position is shown for the $MPV_{Hole\;depth}$ measurement.

To compute Equation 4 coefficients, paired acquisitions of the CDRAD and the LC phantom were performed on Unit A for each beam quality described in Table 2. The theoretical $SC_{CDRAD}$ for each hole depth was computed from Section 2.2.1. From the corresponding LC images, $CNR_{LC}$ was measured using ROIs delimited on the background and on hole depths located in the central module, minimizing the influence of peripheral geometric distortion and ensuring that the calculated values represent detector performance. A linear relationship between $CNR_{LC}$ and hole depth was obtained by regression. CNR uncertainty was minimized using a fully inscribed 800‐pixel ROI, standardized acquisition conditions, and repeated background sampling, which together ensured stable mean signal and noise estimates. As in the CDRAD, two values of $MPV_{\mathrm{Hole}\;\mathrm{depth}}$ and five values of $STD_{\mathrm{Background}}$ were computed for each hole depth. Figure 4 illustrates ROI positioning on the LC phantom.

To ensure consistency in the comparison between the CDRAD and the proposed LC phantom, the analysis was restricted to the 8‐mm‐diameter column of the CDRAD matrix. By fixing the targets’ diameter and varying only the depth, the measured CNR and corresponding SC values reflect contrast–detail differences and minimize the influence of geometric resolution effects.

Substituting Equation (4) into Equation (3) yields a direct relationship between $SC_{CDRAD}$ and $CNR_{LC}$, allowing SC‐equivalent values to be directly obtained from measured CNR values in the proposed phantom.

#### 2.2.3 Clinical validation of the LC phantom

The final validation stage qualitatively assessed agreement between the LC phantom and the CDRAD, focusing on whether visually detectable hole depths were consistent under equivalent exposure conditions.

Three medical physicists and two radiologists subjectively evaluated the low‐contrast resolution threshold for each image. All observers had extensive experience. They identified the shallowest hole depth that could be visually discerned by answering the question: “What is the last hole you can still reliably visualize?”. The corresponding contrast threshold was then recorded for each phantom across multiple fluoroscopy systems and clinically relevant beam qualities. All assessments were conducted within the radiology department, using the same monitor described in Section 2.2.1, under standard illumination conditions (under 75 lux),^24^ with no zoom, image enhancement, or windowing techniques (including window width or window level adjustments) applied. To quantitatively evaluate the agreement between the LC phantom and the CDRAD phantom, paired comparisons of visually determined hole depth threshold values were performed using the Wilcoxon signed‐rank test, with a significance level of 0.05.

Once the agreement between the limiting negative‐contrast thicknesses and the corresponding CNR values was confirmed both quantitatively, as demonstrated in the previous section, and qualitatively, as assessed in the present section for all beam qualities, the SC scale of the CDRAD phantom was adopted as the SC scale for the proposed phantom. Accordingly, SC‐equivalent values for the LC phantom were calculated directly from the measured CNR values using the relationships defined in Equations (3) and (4). The resulting values were directly compared with the simulated SC threshold values obtained from the CDRAD.

Subsequently, the proposed phantom was evaluated on three additional fluoroscopy systems, denoted as Units B, C, and D. Units A and B corresponded to General Electric® OEC One mobile fluoroscopy systems, allowing an assessment of reproducibility across two identical models. Unit C consisted of a SIEMENS® COMPACT SIREMOBIL mobile system, contributing an additional manufacturer and detector configuration to the evaluation. Finally, Unit D was a fixed angiographic system (SIEMENS® Artis Zee), providing comparison with a high‐performance imaging chain.

For this multi‐system phase, observers identified the last visually detectable hole depth for each fluoroscopy unit under beam quality Q1. The corresponding threshold SC values were obtained by applying the previously established SC–hole depth relationship performed with Unit A.

All systems were verified to be fully compliant with established QC standards prior to evaluation. The same five observers assessed LC resolution across all units under identical viewing conditions.

To ensure consistent dosimetric characterization across all evaluated fluoroscopy systems, air kerma rate measurements were obtained at the phantom surface using a properly calibrated RTI® Piranha multimeter.

The authors declare that a large language model (LLM), specifically Google Gemini, was used exclusively for language editing and orthographic revision. The AI tool was not used for the generation, analysis, or interpretation of scientific content. All scientific aspects of the work were developed independently by the authors, who assume full responsibility for the manuscript.

## 3 RESULTS

### 3.1 Correlation between SC and CNR obtained with the CDRAD phantom

**FIGURE 5:**
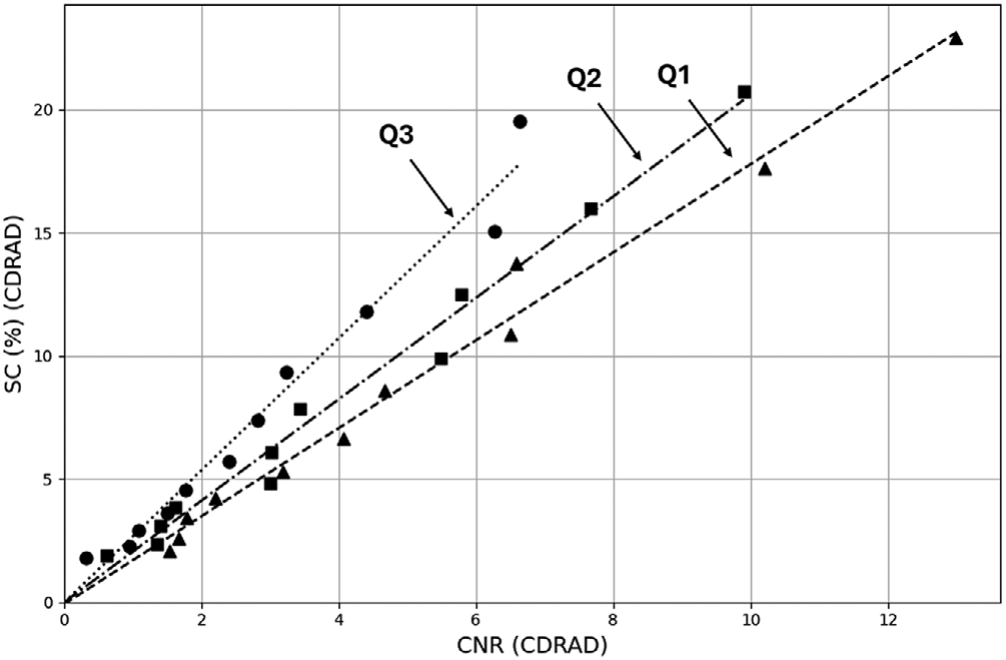
SC as a function of $CNR_{CDRAD}$ for the beam qualities described in Table 2. Linear regressions were: Q1: *y* = 1.787x – 0.086 (*R* ^2^ = 0.986); Q2: *y* = 2.062x ‐ 0.011 (*R* ^2^ = 0.984); Q3: *y* = 2.683x ‐0.016 (*R* ^2^ = 0.976).

The SC values calculated for each hole depth are presented in Figure 5 as a function of the measured $\mathrm{CN}R_{\mathrm{CDRAD}}$.

A strong linear relationship is observed for all beam qualities, in which progressively lower SC values are associated with thinner targets and correspondingly lower CNR values. The fitted linear intercepts were found to be close to zero, as expected for a physically consistent system.

### 3.2 Calculating SC values from the LC phantom

**FIGURE 6:**
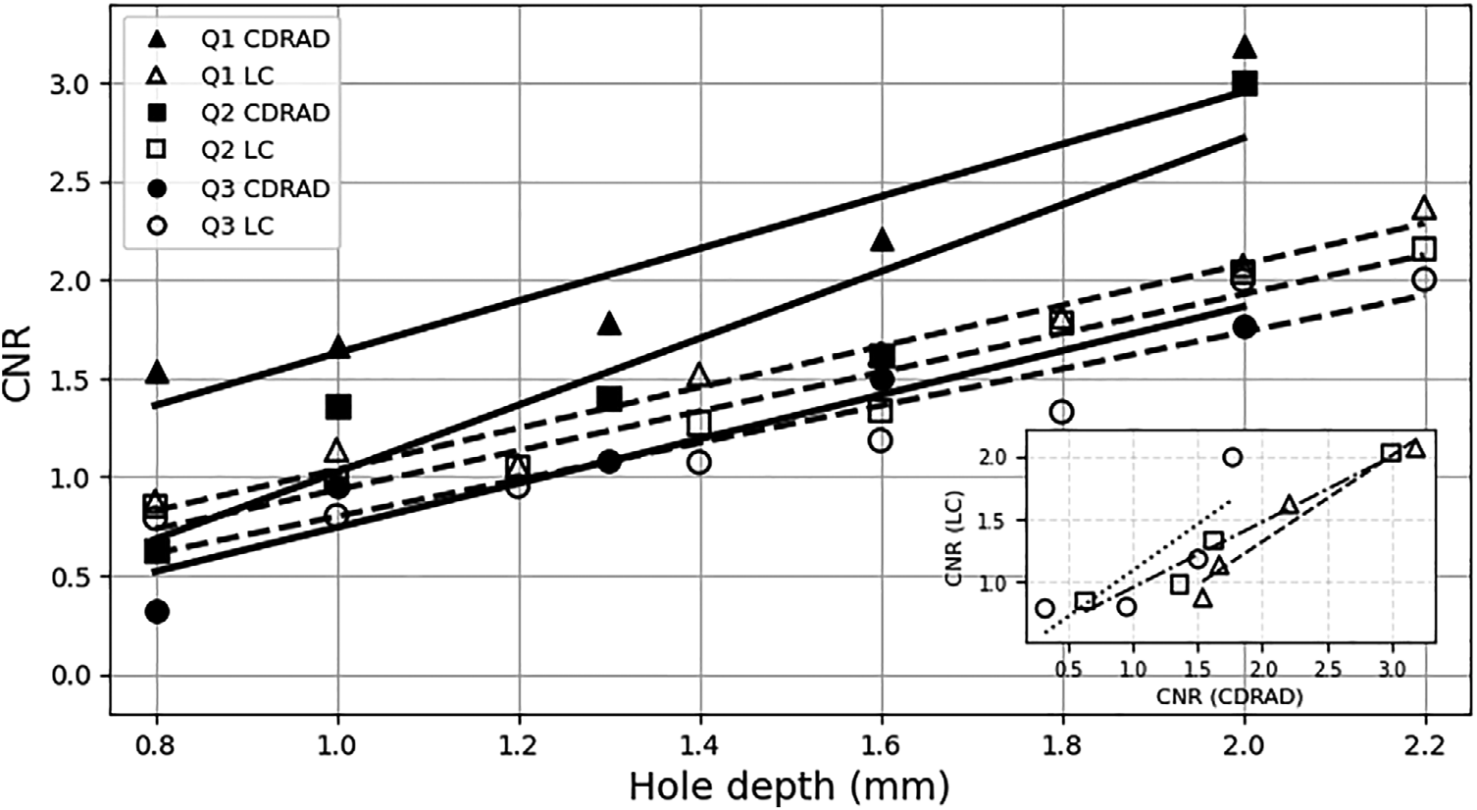
CNR as a function of hole depth for the three beam qualities using the LC phantom and the CDRAD phantom. Linear regressions were: Q1 CDRAD: *y* = 1.326x + 0.301 (*R* ^2^ = 0.894); Q1 LC: *y* = 1.040x – 0.003 (*R* ^2^ = 0.966); Q2 CDRAD: *y* = 1.696x – 0.673 (*R* ^2^ = 0.870); Q2 LC: *y* = 0.992x – 0.057 (*R* ^2^ = 0.954); Q3 CDRAD: *y* = 1.119x – 0.375 (*R* ^2^ = 0.920); Q3 LC: *y* = 0.934x – 0.135 (*R* ^2^ = 0.882). Linear regressions shown in the inset were: Q1: *y* = 0.686x ‐ 0.048 (*R* ^2^ = 0.943); Q2: *y* = 0.521x + 0.438 (*R* ^2^ = 0.953); Q3: *y* = 0.734x + 0.361 (*R* ^2^ = 0.693).

The CNR values calculated for each hole depth for both phantoms are shown in Figure 6. The insets show the relationship between $CNR_{LC}$ and $CNR_{CDRAD}$ for the three investigated beam qualities.

The air kerma rates measured at the entrance of the LC phantom were 73, 78, and 66 µGy/min for beam qualities Q1, Q2, and Q3, respectively. The corresponding tube currents were 0.4, 0.2, and 0.1 mA. Given the proportional relationship between air kerma rate and tube current, these settings contribute to the differences observed among beam qualities.

Notably, a slightly different slope was obtained for the LC phantom, indicating a reduced sensitivity of CNR to small variations in hole depth. This feature reflects intrinsic differences between the devices, suggesting a more gradual and stable response for the proposed phantom. Importantly, the same depth‐dependent trend was preserved across all beam qualities, supporting the suitability of the proposed phantom for quantitative low‐contrast resolution assessment under clinically relevant fluoroscopic conditions.

**FIGURE 7:**
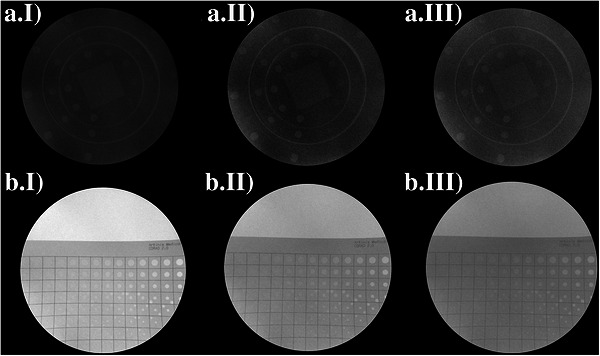
Paired fluoroscopic acquisitions obtained with the proposed LC phantom (panels a) and the CDRAD phantom (panels b) for the three investigated beam qualities. For each beam quality described in Table 2, the corresponding image pairs (a.I/b.I, a.II/b.II, and a.III/b.III, respectively) were acquired under identical exposure conditions.

Figure 7 presents the paired fluoroscopic acquisitions obtained with the proposed LC phantom (panels A) and the CDRAD (panels B) for the three investigated beam qualities described in Section 2.2.1, using Unit A. For each beam quality defined in Table 2, the corresponding image pairs (A.I/B.I, A.II/B.II, and A.III/B.III, respectively) were acquired under identical exposure conditions and constituted the dataset used for the subjective evaluations. For clarity of presentation, the images shown in Figure 7 were displayed using optimized window/level settings; the original, unaltered images, in the same conditions described in Section 2.2.1, were used for all subjective assessments.

**FIGURE 8:**
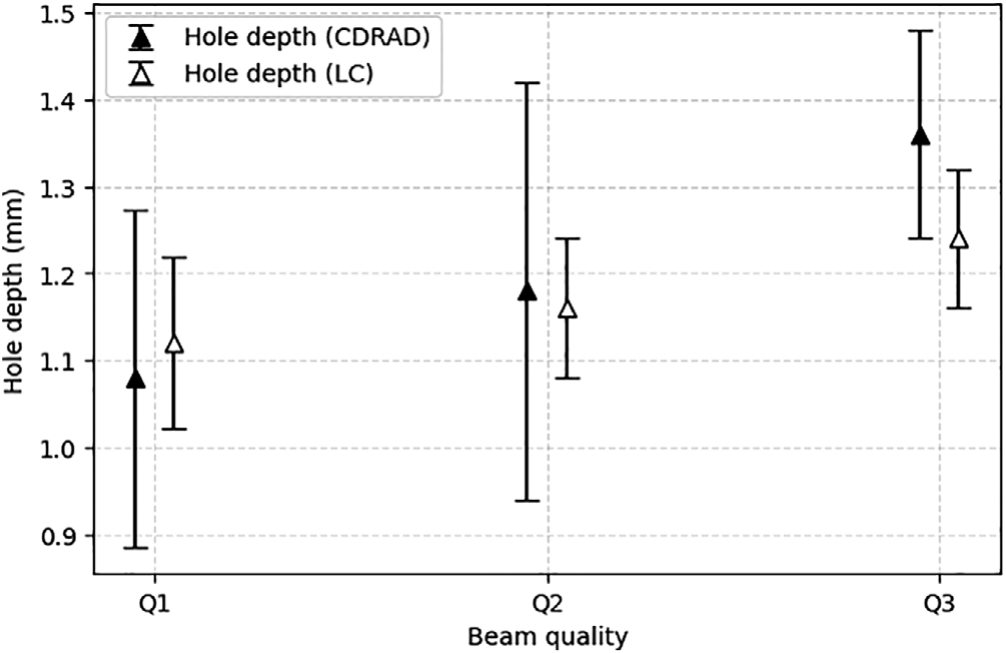
Observer‐based results for the limiting hole depth identified with both phantoms using the fluoroscopy images shown in Figure 7.

Figure 8 shows the mean limiting hole depth from the independent responses of the five observers (Section 2.2.3). Because mean values are reported, intermediate values that do not correspond to discrete hole depths are expected. Error bars indicate the standard deviation, reflecting interobserver variability.

The 95% confidence intervals for the hole depths thresholds across beam qualities Q1–Q3 were 0.80–1.36, 0.87–1.49, and 1.19–1.53 for the CDRAD phantom, and 0.99–1.25, 1.04–1.28, and 1.12–1.36 for the proposed LC phantom, respectively. The paired Wilcoxon signed‐rank tests demonstrated no statistically significant differences between phantoms in terms of threshold hole depth (Q1: *p* = 0.8125; Q2: *p* = 0.8125; Q3: *p* = 0.1250) for any of the evaluated beam qualities.

**FIGURE 9:**
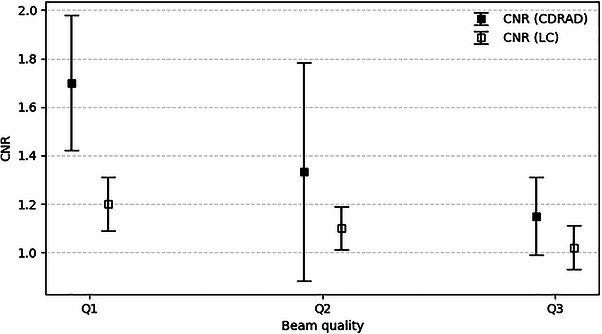
Observer‐based results for the limiting CNR identified with both phantoms using the fluoroscopy images shown in Figure 7.

Correspondingly, Figure 9 presents the mean CNR values associated with the threshold hole depths, with error bars indicating the corresponding standard deviations.

The 95% confidence intervals for the CNR thresholds across beam qualities Q1–Q3 were 1.38–2.09, 0.89‐1.77, and 0.98–1.21 for the CDRAD phantom, and 1.04–1.29, 0.98–1.21, and 0.92–1.13 for the proposed LC phantom, respectively. The LC phantom demonstrated improved detectability (lower CNR values for similar threshold hole depths), particularly in Q1.

In both Figures 8 and 9, the standard deviation values observed for each beam quality reflect a high degree of consistency in threshold identification among observers. Moreover, the progressive increase in the visually identified limiting hole depth, accompanied by a decrease in CNR with increasing beam hardness, is consistent with the expected reduction in subject contrast at higher beam energies.

**FIGURE 10:**
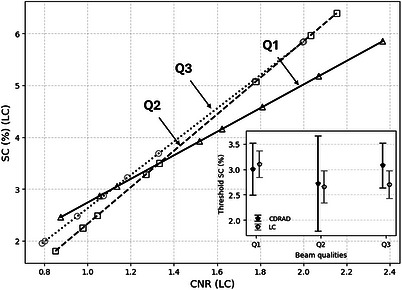
SC values for the LC phantom calculated from CNR measurements. Linear regressions were: Q1: *y* = 2.278x + 0.459; Q2: *y* = 3.524x − 1.197; and Q3: *y* = 3.214x − 0.588. In the inset, threshold SC values obtained for the CDRAD phantom and LC phantom.

Given the agreement between qualitative and quantitative evaluation metrics across beam qualities, the formulation defined at Section 2.2.2 was adopted as the quantitative mapping used to derive SC‐equivalent values for the proposed LC phantom. Based on this formulation, Figure 10 was obtained. The inset shows the threshold SC values calculated using the linear fits derived in Section 3.1 and threshold CNR values from Figure 9.

The consistency of threshold SC values across different spectral conditions indicates that the proposed phantom provides a reliable and equivalent assessment of low‐contrast performance over a broad range of beam qualities. In addition, the standard deviation of SC thresholds obtained for the LC phantom were systematically lower than those observed for the CDRAD phantom. This reduced variability reflects not only its intrinsic response but also the finer depth discretization implemented in the proposed design, which enables a more precise identification of the visually limiting target.

### 3.3 Clinical validation of the LC phantom

Table 3 summarizes the SC threshold values obtained during the clinical validation of the LC phantom, organized by imaging unit and beam quality. For each fluoroscopy system, the table reports the nominal II or flat‐panel size, the measured air kerma rate at the LC phantom surface, and the corresponding SC thresholds derived from the visual assessments for both phantoms.

**TABLE 3:** Nominal detector sizes, air kerma rate measurements and SC threshold values between LC and CDRAD phantoms for exposure conditions using Units B, C and D.

Unit	Beam quality	Nominal II / flat‐panel size (cm)	Air Kerma Rate (at LC phantom surface) (µGy/min)	SC threshold visual assessment (LC)	SC threshold visual assessment (CDRAD)
B	Q1	22.0	66	3.20 ± 0.21	3.39 ± 1.00
C		22.0	78	3.10 ± 0.26	3.20 ± 0.70
D		42.0	73	3.39 ± 0.21	3.71 ± 0.43

The visual SC thresholds obtained with the proposed LC phantom demonstrated consistent behavior across different fluoroscopy systems and beam qualities, remaining in close agreement with those derived from the CDRAD phantom. The reported standard deviations, reflecting interobserver variability among the five independent evaluators, indicate limited dispersion in threshold identification. Differences observed between units fall within the expected range for heterogeneous imaging systems and visual assessments, while preserving the same order of magnitude and overall low‐contrast detectability trends.

## 4 DISCUSSION

A key motivation for developing the LC phantom was to provide an accessible and reproducible alternative to commercially available low‐contrast phantoms that may present logistical or financial constraints in some clinical environments. The phantom can be fabricated locally using widely available materials and standard 3D printing. Although the direct fabrication cost was approximately USD 35 in raw materials, with an additional investment of about USD 700 for the 3D printer (excluding taxes), this estimate does not include infrastructure or personnel time. The adopted methodological structure aligns with recent recommendations for phantom‐based studies, promoting reproducibility in the design, documentation, and quantitative evaluation of 3D‐printed test devices.^25^

The strong linear relationship observed between theoretical SC and image‐derived CNR (Figure 5) across all investigated beam qualities (R^2^ ≈ 0.98) is a key finding of this study. CNR provides a robust link between spectral modeling and fluoroscopic image data, with near‐zero intercepts supporting the internal consistency of the approach. When evaluated as a function of hole depth, both phantoms exhibited the expected monotonic behavior (Figure 6). The linear correspondence between values obtained with the LC phantom and those measured with the CDRAD phantom further demonstrates quantitative traceability.

Despite differences in CNR–depth slopes, subjective visual assessments showed no statistically significant differences in threshold hole depth values between devices across all beam qualities. The resulting SC thresholds, for all units and beam qualities, remained within the clinically expected 2–4% range required by international protocols^17^ (Figure 10). The finer 0.2‐mm depth increments of the LC phantom, compared with the 0.3‐mm steps in the CDRAD, provide a more refined discretization of the evaluated contrast range, which is reflected in the lower standard deviation observed in the subjective assessments. The reduced correlation observed for the Q3 beam quality is consistent with lower intrinsic contrast at higher energies and does not compromise overall validity. Extending the evaluation to additional beam qualities would allow validation under more diverse operating conditions, further reinforcing the clinical applicability and generalizability of the proposed framework.

Clinical validation across multiple fluoroscopy systems, including mobile and fixed units from different manufacturers, demonstrated consistent low‐contrast thresholds, supporting cross‐platform robustness (Table 3). Compared with low‐contrast tools that relied primarily on subjective assessment, the present study integrates quantitative image‐quality metrics and spectral modeling within a unified framework while preserving practical clinical applicability. The selected beam qualities reflect common clinical fluoroscopy settings. The phantom was therefore evaluated under representative conditions, supporting the robustness and relevance of the proposed framework.

Previous studies explored the relationship between quantitative image‐quality metrics and patient exposure using standardized phantoms, demonstrating that variations in detector performance or dose settings are not always linearly correlated with clinically perceptible image improvements.^26^ In this context, combining quantitative CNR analysis with structured visual threshold assessment provides a clinically meaningful evaluation strategy. Future investigations incorporating formal inter‐printer reproducibility assessment, independent characterization of material attenuation properties, evaluation of additional depth–diameter combinations, and systematic analysis of post‐processing variability across fluoroscopy systems may further strengthen the generalizability of the proposed framework.

Beyond its immediate application, this study supports the integration of locally fabricated 3D‐printed phantoms into quantitatively traceable and clinically interpretable QC frameworks while maintaining comparability to a single reference device.

## 5 CONCLUSION

The developed LC phantom provides a reliable and quantitatively validated tool for evaluating low‐contrast performance in fluoroscopy. Quantitative agreement and consistent qualitative performance were observed relative to CDRAD across the examined beam qualities. These results support 3D printing as a viable approach for structured phantom fabrication. Open and reproducible design frameworks for phantoms may further improve methodological consistency in image‐quality assessment across diverse clinical environments.

## AUTHOR CONTRIBUTIONS

**Túlio Guilherme Soares Marques**: Conceptualization; phantom design and fabrication; experimental work; data analysis; validation; and manuscript drafting. **Diana Pina**: Scientific supervision; methodological guidance; critical review; and manuscript revision.

## CONFLICT OF INTEREST STATEMENT

The authors have no relevant conflicts of interest to disclose.

## Supporting information




Supporting Information

**Supporting File 1**: acm270650‐supp‐0002‐SuppMat.docx.
