# Supplementary material for: Development and validation of a 3D printed phantom for image quality assessment in fluoroscopy
Source: J Appl Clin Med Phys. 2026 Jun 4;27(6):e70650. doi: 10.1002/acm2.70650 (PMC13238637; doi:10.1002/acm2.70650)
Supplement: Supplementary file 2 — Supporting File 1: acm270650‐supp‐0002‐SuppMat.docx. [file ACM2-27-e70650-s002.docx]

**Supplementary material**

**Supplementary File S-1 – Access to LC phantom STL Model**

Supplementary File S-1 containing the STL files required to reproduce the phantom described in this study is available through the journal supplementary material and has also been deposited in the Zenodo public scientific repository under restricted access (<https://zenodo.org/records/18827666> ). The file will be made fully publicly available upon acceptance and publication of this manuscript.
